# Testosterone and testicular changes in F1 offspring of Wistar rats maternally exposed to nicotine during gestation

**DOI:** 10.5935/1518-0557.20180031

**Published:** 2018

**Authors:** Ibukun P Oyeyipo, Damilare H Adeyemi, Temilade R Abe

**Affiliations:** 1 Department of Physiology, College of Health Sciences, Osun State University, Osogbo, Osun State, Nigeria

**Keywords:** Nicotine, pregnancy, histology, testosterone, testes, epididymis

## Abstract

**Objectives:**

This study aimed to determine the effect of intrauterine exposure to nicotine in the first fourteen days of gestation on the testicular function of male Wistar rats.

**Methods:**

Pups of both control and nicotine-treated groups were selected and sacrificed on day 60 after birth. Birth weight, weight of reproductive organs, hormonal profile, and histopathology were determined in the first filial (F1) generation.

**Results:**

Significant decreases in birth weight and litter size were found in the pups treated with nicotine when compared with the animals in the control group. Significant decreases were also observed in the testicular weight of nicotine-treated rats, but not in epididymal weight, when compared to controls. Testosterone levels were significantly decreased, atrophy was observed in the genital epithelial cells, and distortions were noted in the testes of nicotine-treated F1 males.

**Conclusion:**

These results suggest that nicotine exposure during pregnancy may cause endocrine disruption, and thus produce deleterious effects on offspring reproductive function.

## INTRODUCTION

Much has been documented on the detrimental effects of smoking on human health. Smoking during pregnancy has been associated with numerous obstetrical, fetal, and developmental complications, as well as increased risk of adverse health consequences in the adult offspring (Haustein, 1999). Exposure to cigarette smoke is a great burden on global health and has significant impact on the well-being, with growing concerns on various systemic functions including reproduction. The World Health Organization (WHO) estimated that smoking affects approximately a third of the world's population; some 80,000-100,000 children begin smoking every day, and half of the ones who start smoking at younger ages are projected to go on to smoke for 15 to 20 years (WHO, 1999). Surprisingly, despite anti-smoking efforts and campaigns, smoking rates are still high and about 50% of all smokers continue to smoke after becoming pregnant (Alshaarawy & Anthony, 2015). Studies have also estimated that at least 12% of the infants born in the United States are exposed prenatally to maternal smoking (Tong *et al*., 2013). Intrauterine exposure to tobacco smoke has been identified as the most significant preventable cause of decreased physiological function at birth and throughout life (Barker, 1998; Dietz *et al*., 2010).

Tobacco combustion yields about 4000 compounds; smoke can be divided into a gaseous phase and another phase made of particles. The principal harmful components are carbon monoxide, nitrogen, oxide, ammonia, and volatile hydrocarbons with the main component of the particle phase being nicotine. Nicotine is a psychoactive substance, and is one of the few natural liquid alkaloids. It is also one of the most heavily used addictive drugs in the United States (WHO, 1992). Each cigarette contains about 8-20 milligrams of nicotine, but only about 1-2 milligrams end up being taken in since not all of the puff is absorbed in the lungs.

Several studies demonstrated that cigarette smoking is associated with decreases in the fertilization rate of couples undergoing in-vitro fertilization (Haustein, 1999). In addition, other authors have reported that nicotine affects fertility rates (Oyeyipo *et al*., 2011), semen volume (Pasqualotto *et al.*, 2006), sperm concentration and motility (Oyeyipo *et al*., 2011; Vine, 1996), as well as hormonal profile (Oyeyipo *et al*., 2013). Nicotine in cigarette smoke increases the rate of follicular destruction and accelerates the loss of reproductive function (Mai *et al*., 2014). Lifestyle factors impact human development at any stage (pre-natal, adolescence and adulthood) and may mediate mechanisms that disturb the morphologic, endocrine, antioxidant or fertilizing capacity of the reproductive system (McMillen & Robinson, 2005). It is also clear in the concept of fetal programming that exposure to uterine challenges affects the phenotype of the offspring through lifelong gene expression patterns initiated during critical developmental stages. These phenotypes may subsequently affect future generations (Barker, 1998; Nyirenda *et al*., 1998).

Few studies in the literature have reported effects of in-vitro and intrauterine nicotine exposure and indicated that nicotine concentration at certain doses causes miscarriage within the first seven days of gestation (Lambers & Clark, 1996). However, no study has documented the reproductive effect of nicotine exposure during other gestational periods.

In spite of the growing knowledge on the adverse effects of nicotine on reproduction, the generational reproductive outcomes relating to testicular function in litters due to maternal exposure during pregnancy have not been investigated. This study was therefore designed to investigate the effects of nicotine administration in the first fourteen days of gestation on the testicular function of the first filial generation (F1) of the male offspring of Wistar rats, with a view of giving an insight to programming effects and generational outcomes of offspring delivered by animals exposed to nicotine.

## MATERIALS AND METHODS

### Animals and treatment

Eighteen mature nulliparous female rats (12 weeks) with body weights ranging between 150-180g were included in the study. Fertile male animals of the same age and weight were cohabited for mating. The animals were procured and kept in the vivarium of the College of Health Sciences at Osun State University, Nigeria. The animals were housed individually in cages, fed with standard pellet diet, and offered water ad libitum. Throughout the experiment, the animals were maintained on a 12-hour light/12-hour dark cycle under constant room temperature. Pairing for mating was 1:1 for males/females. Mating was confirmed by the presence of a sperm-positive vaginal smear or a copulation plug in the females. The day after which either was found was considered as day 1 of gestation. The pregnant rats were randomly assigned to three groups, as follows: Group I - six (6) rats given 0.2 ml/kg of normal saline solution throughout the gestational period; Group II - six (6) rats given nicotine (1.0mg/kg) orally on gestation days 1-7; Group III - six rats given nicotine (1.0mg/kg) orally between on gestation days 7-14. After delivering their offspring, the animals had the following parameters measured: body weight, absolute and relative reproductive organ weight, serum testosterone, and histopathology of the testes from 10 randomly selected F1 males per experimental group on day 60.

### Blood sample collection

On day 60, the animals were sacrificed by anestesia using chloroform; blood was collected from each animals via cardiac puncture with a 2-ml syringe needle and placed in a tube with plain serum for hormonal analysis. The samples were centrifuged at 3000 rpm for 15 minutes. The serum was used to analyze the level of testosterone.

### Organ collection and histology

The animals were dissected and the organs of interest (testes, epididymes, seminal vesicle, prostate, liver, kidney, and spleen) were removed, cleared of adherent tissues and weighed immediately on an electronic scale model DT 300 with a capacity of 0.01-300g. The organs were fixed in Bouin's solution for six hours, transferred to 10% Formalin, sectioned and stained routinely with hematoxylin and eosin for microscopy studies. Stained slides were cleared in xylene before they were mounted on the microscope for histological examination. Photomicrographs of the slides were then taken.

### Serum assay for testosterone

An enzyme-based immunoassay system was used to measure testosterone levels in the serum samples obtained. The EIA kit was acquired from Immunometrics (London, UK) and contained the respective EIA enzyme label, EIA substrate reagent and EIA quality control sample. Quality control runs were carried out at the beginning and at the end of the assay to ascertain the acceptability with respect to bias and batch variation.

### Statistical Analysis

The data obtained were presented as mean ± SEM for each group. Student's test was used to assess the presence of significant differences between groups. Differences with *p*<0.05 were deemed significant. All statistical comparisons and tests were performed using SPSS (SPSS Inc., Chicago, IL, USA) for Windows.

## RESULTS

### Effects of maternal exposure to nicotine on body weight and litter size in F1 male pups

The birth weight and the final body weight of the control and nicotine-treated animals are shown in Table 1. The body weight of the animals treated with nicotine after 60 days was 91.88±3.73, while controls after 60 days weighed 98.36±1.43. There was no significant difference in the growth rates of control and nicotine-treated rats.

**Table 1:** Body weight and litter size of F1 male pups from rats treated with nicotine at different gestational periods

Groups	Litter size	Birth weight (g)	Final Weight (g)	Absolute Difference (g)
**Control**	7.80±0.40	6.37±0.49	98.36±1.43	91.99±1.26
**GD 1-7**	0.00±0.00	0.00±0.00	0.00±0.00	0.00±0.00
**GD 7-14**	5.02±0.26*	5.04±0.16*	91.88±3.73	86.84±1.32

Data are expressed as mean ± SEM (*n*=10).

* *p*<0.05: compared to Controls. *GD; Gestation day.*

### Effects of maternal exposure to nicotine on the testes and epididymes of F1 male pups

A significant decrease (*p*<0.05) was observed in the mean weight of the testicles of the treated rats when compared to controls; the mean weight of the epididymis of treated rats was comparable to that of controls, as shown in Table 2. Intrauterine exposure to nicotine for 14 days did not yield any significant effect on the weight of the visceral organs (prostate, seminal vesicle, kidney, spleen, and liver), as shown in Table 3.

**Table 2:** Reproductive organ weight of F1 male pups from rats treated with nicotine at different gestational periods

Groups	Testicular weight (g)	Epididymal weight (g)	Relative Testicular Weight (%)	Relative Epididymal weight (%)
**Control**	0.36±0.04	0.07±0.02	0.37±0.03	0.07±0.02
**GD 7-14**	0.21±0.01*	0.05±0.01	0.22±0.02*	0.05±0.02

Relative testicular weight=Testicular weight/Body weight×100, Relative epididymal weight=Epididymal weight/Body weight×100. Data are expressed as mean ± SEM (*n*=10).

* *p*<0.05: compared to Control, *GD; Gestation day*

**Table 3:** Visceral organ weight in F1 male pups from rats treated with nicotine at different gestational periods

Groups	Prostate	Seminal vesicle	Liver	Kidney
**Control**	0.11±0.02	0.29±0.06	2.19±0.04	0.63±0.05
**GD 7-14**	0.11±0.01	0.28± 0.07	2.10±0.05	0.61±0.04

Data are expressed as mean ± SEM (*n*=10), *GD; Gestation day.*

### Effects of maternal exposure to nicotine on serum testosterone in F1 males

**Figure 1:**
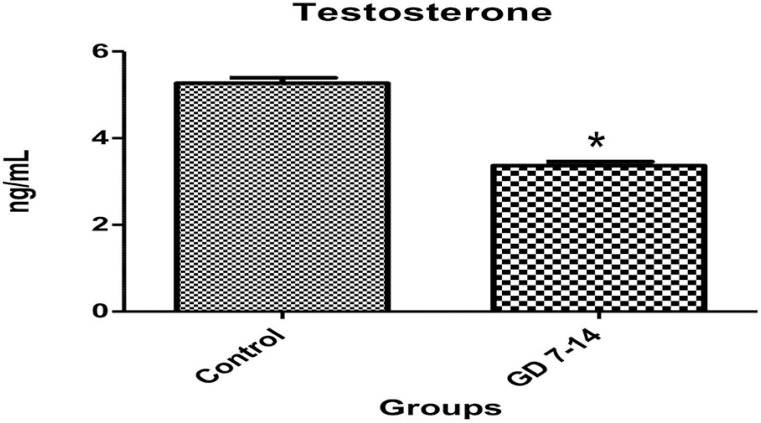
Effect of nicotine on experimental groups. Data are expressed as mean ± SEM (*n*=10). *p<0.05: compared to Controls, GD; *Gestation day*.

There was a significant decrease (*p*<0.05) in serum testosterone levels in the offspring of treated rats when compared to the offspring of control rats (Figure 1).

### Effects of maternal exposure to nicotine on the histology of testes and epididymes in F1 males

**Figure 2:**
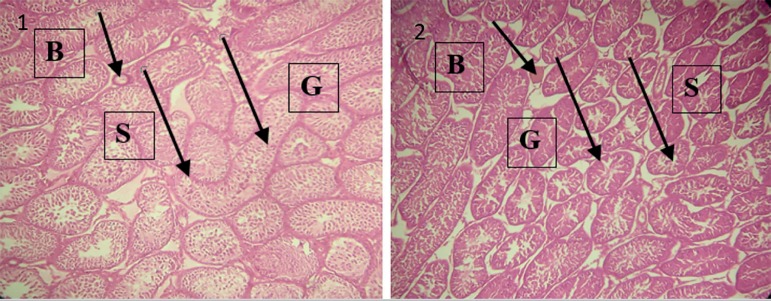
Plate 1- Photomicrograph showing the testes of control pups with normal seminiferous tubules and distinct peritubular tissue (S), with intact and adequate germ cells and spermatids (G). Blood vessels are also present (B). Plate 2- Photomicrograph showing the testes of nicotine-treated pups with apparently atrophic and indistinct seminiferous tubules (S). It also shows gradual erosion of the germ cell layer (G). Blood vessels are also present (B).

The histology sections of testicular tissue of control animals had normal histological architecture. The seminiferous tubules and peritubular tissues were distinct; germ cells and spermatids were intact and adequate, indicating that the tissues were essentially normal. However, the offspring of nicotine-treated rats between gestation days 7-14 had testicular tissue with obviously distorted cytoarchitecture. The seminiferous tubules appeared to be atrophic, there was marked loss of spermatids, and gradual erosion of the germ cell layer, as shown in Figure 2.

## DISCUSSION

Some isolated effects of nicotine on the reproductive system have been studied in both male and female humans and animals (Patterson *et al.*, 1990; Winders & Grunberg, 1990; Oyeyipo *et al*., 2013), but no information is available in literature on the effects of nicotine on the testicular function of the F1 male offspring of female rats treated with nicotine in the first fourteen days of gestation. Nicotine was given by gavage in this study, which is in agreement with the possible route of human exposure during gestation; the dosage used in this study emulates heavy smoking.

In the present study, the effects of nicotine on the testicular function of F1 Wistar rats showed decreases in testosterone associated with abnormal histology of the testes. It is worth noting that the administration of 1.0mg/kg of nicotine in the first seven days of pregnancy led to miscarriage in all animals; none was able to deliver offspring as also reported previously in another study (Omotoso *et al.*, 2013). The male offspring in our study also had significant decreases in birth weight and testicular weight. Nicotine is known to affect the gestational age of fetuses at the time of parturition and their growth rate, which determine the birth weight of the pups (Lambers & Clark, 1996). Decreases in body weight were observed at the time of birth and when the animals were slaughtered. Decreases in birth weight might be a result of loss of appetite and reduced food intake caused by nicotine during pregnancy (Jo *et al*., 2002; Chen *et al*., 2012). Low birth weight resulting from disproportionate fetal growth has been listed as a major risk factor implicating the occurrence of fetal programming (Berthold, 2007). However, other authors have dismissed the association between some programming and birth weight (Nathaniesz & Thornburg, 2003).

The role of hormones as a programming signal has been reported in humans and experimental studies (Fowden *et al*., 2006). Although nicotine has been previously reported to decrease testosterone when administered orally to male rats (Oyeyipo *et al*., 2013), our study found for the first time that there was also a significant reduction in the serum testosterone levels of the male F1 offspring of nicotine-treated females. This is an indication that it might alter the hypothalamic-pituitary-gonadal axis function in the offspring, thereby adversely affecting puberty and other reproductive functions of the offspring throughout their life. Testosterone is necessary for the development and normal functioning of the testes and other male accessory reproductive glands. Low serum testosterone levels have been reported to adversely affect the structure, weight, and function of the testes and epididymes (George & Wilson, 1986; Mooradian *et al.*, 1987). Hence, the significant reduction in weight of the testes might be associated with the decrease in the serum level of testosterone of the offspring of treated rats, while the decrease in serum testosterone level of rats exposed to nicotine might have resulted from the disruption of testicular cytoarchitecture with nicotine adversely affecting the number of Leydig cells responsible for testosterone synthesis (Oyeyipo *et al*., 2010).

In conclusion, these results suggest that nicotine decreases serum testosterone and introduces deleterious effects on male reproductive organs and in the histology of the offspring of nicotine-treated rats. The observed hormonal imbalances and distortion of testicular cytoarchitecture might affect puberty and other reproductive functions in adulthood. Further studies are required to explore the possible mechanisms tied to hormonal imbalances.
